# Physical fitness mediates the inverse association between fatness indicators and academic achievement, despite the school vulnerability of adolescents—The Cogni-Action Project

**DOI:** 10.3389/fnut.2022.904831

**Published:** 2022-10-26

**Authors:** Guillermo Gajardo-Araya, Sam Hernández-Jaña, Jorge Olivares-Arancibia, Gerson Ferrari, Pedro Delgado-Floody, Carlos Cristi-Montero

**Affiliations:** ^1^Magíster en Educación, Mención Política y Gestión Educativa, Facultad de Filosofía y Humanidades, Universidad Austral de Chile, Valdivia, Chile; ^2^IRyS Group, Physical Education School, Pontificia Universidad Católica de Valparaíso, Valparaíso, Chile; ^3^Grupo AFySE, Investigación en Actividad Física y Salud Escolar, Escuela de Pedagogía en Educación Física, Facultad de Educación, Universidad de Las Américas, Santiago, Chile; ^4^Escuela de Ciencias de la Actividad Física, el Deporte y la Salud, Universidad de Santiago de Chile (USACH), Santiago, Chile; ^5^Department of Physical Education, Sport, and Recreation, Universidad de La Frontera, Temuco, Chile

**Keywords:** mediating factor, exercise, abdominal obesity, poverty, socioeconomic factor

## Abstract

**Objective:**

This study aims to determine the mediating role of physical fitness in the relationship between fatness indicators and academic achievement, exploring the influence of school vulnerability.

**Methods:**

A total of 1,296 Chilean adolescents (aged 10 to 14 years; 50% girls) participated in this study. The global fitness score (GFS) was obtained by adding the three main components of the ALPHA fitness test: cardiorespiratory fitness (CRF), muscular fitness (MF), and speed/agility fitness (SAF). CRF was evaluated through the 20 m shuttle run test; MF by upper and lower limb strength tests; and SAF by the 4 × 10 shuttle run test. BMIz and WHtR were evaluated as general (unspecific) and central (specific) fatness indicators. Academic achievement was established through grades in math, language, and science and their average scores. Multiple mediation analyses were performed according to two models, adjusted for sex, maturity, and schools (model 1), and in model 2, the school vulnerability index (SVI) was added. The SVI is an important proxy of socioeconomic status at the school level, and it was categorized as high-, mid-, or low-SVI. Mediation percentages were calculated, and confidence intervals (bootstrapping) were used to establish significant findings.

**Results:**

CRF, SAF, and GFS mediate the relationship between fatness indicators and academic achievement, both partially and totally (ranging from 12.7 to 59.2%). However, MF did not show any mediation effect. After controlling for SVI, CRF, and GFS, mediation changed from partial to total in the associations between math and science with WHtR. Although SAF contributed to GFS mediation, CRF seems to have the most significant mediation role for all academic achievements, regardless of SVI and the fat indicator studied.

**Conclusion:**

A higher level of general physical fitness, especially CRF, significantly mediates the detrimental influence of fatness on the academic achievement of schoolchildren. This study suggests that physical fitness plays a relevant role in academic and public health, considering the high prevalence and detrimental influence of obesity and school vulnerability in children and adolescents.

## Introduction

The detrimental impact of obesity on a variety of metabolic and brain health indicators in childhood is being widely addressed (e.g., cardiometabolic disease, cognitive functioning, brain health) (1, 2), and, simultaneously, it has been related to other important collateral indicators, such as academic achievement (3). For instance, some studies have shown differences in gray and white matter brain regions according to children’s weight status (4), which might affect their cognitive and, in turn, academic achievement (5–7).

In general, evidence has established the inverse relationship between fatness indicators and academic achievement (8–12). Most studies in this research discipline have evaluated fatness using body mass index (BMI), a valuable, easy, and fast method to determine nutritional status (13). However, this is a non-specific method related to fat distribution. The location of adiposity in the body (i.e., peripheral or central) is essential due to its impact on low-grade inflammation (14). In this sense, an indicator of central adiposities, such as the waist-to-height ratio (WHtR), is related to a higher level of inflammation markers, which, in turn, are associated with neurodegeneration and cognitive impairment (15, 16). Therefore, BMI and WHtR might differ in the way they are related to academic achievement. Indeed, a large-scale cross-sectional study of schoolchildren showed that WHtR was more closely related to academic achievement than BMI (14).

Promoting regular physical activity is essential to counteract the impact of fatness on health. This is a low-cost strategy that contributes to the control of childhood overweight and obesity and can improve several parameters of physical fitness, such as cardiorespiratory fitness (CRF), muscular fitness (MF), and speed-agility fitness (SAF), related to brain health and academic achievement (17–19). Regarding the latter, a recent systematic review concluded that adolescents with higher physical fitness levels (mainly CRF and MF) presented better academic achievement (20); however, findings in this field of study are heterogeneous, especially with MF (21) and SAF (22).

Mediation analysis plays a relevant role in finding a suitable statistical approach to study the relationship between fatness, fitness, and academic achievement, particularly the influence of fitness on this relationship. To date, some studies have shown that CRF, MF, and SAF positively mediate the inverse association between fatness and academic achievement (9, 23). However, as the socioeconomic status (SES) background of young people is a root problem linked to inequalities in health (24), brain development (25), and academic achievements (26), exploring the influence of SES background on future mediation approaches seems to be necessary for at least two key reasons. First, most studies adjust their analyses for confounding by SES (i.e., parental education, income, occupational class, and others); however, evidence shows that they are not interchangeable in social epidemiology. Thus, it is strongly recommended to use a more complex indicator of SES (27, 28), such as the school vulnerability index (SVI), a complex indicator involving several social factors based on Chilean school clusters. Second, by 2030, 63% of children worldwide will live in lower-middle-income countries, which presents growing economic difficulties, widening the educational gap between SES groups and nations (29). This way, data and evidence-based on regional contexts (a programmatic approach from UNICEF) are necessary to deal with this global learning crisis that prepares children and adolescents for life, work, and active citizenship.

Therefore, the present study aimed to determine the mediating role of several physical fitness variables in the association between fatness indicators and several academic achievements in adolescents, exploring the influence of school vulnerability.

## Materials and methods

### Study design

This study is part of the Cogni-Action Project (from March 2017 to October 2019), which establishes associations between physical fitness, physical activity, and sedentary life with academic achievement, cognitive performance, and brain structure and function in Chilean schoolchildren (30). This project was retrospectively registered (8/July/2020) with the Research Registry (ID: researchregistry5791) and was approved by the Ethics Committee of the Pontificia Universidad Católica de Valparaíso (BIOEPUCV-H103–2016). This study was prepared according to the STROBE guidelines for cross-sectional studies (31) and the AGReMA Statement for Reporting mediation analyses (32). Before participation, written consent was obtained from the school principal, parents, and participants.

### Study population

The Cogni-Action Project collected information on 1,296 girls and boys (1:1) between 5th and 8th grade (aged 10–14 years) from public, subsidized, and private schools in Valparaiso, Chile. A total of 19 schools participated in the project. More information on the Cogni-Action Project is provided in Solis-Urra et al. (30). It is important to note that this project and study adhere to the definition of adolescence, which is established as a period between 10 and 24 years of age (33). The inclusion criteria for this project were schoolchildren from 5^th^ to 8^th^ grade, while exclusion criteria for this study were based on a lack of data for the following variables: body mass index Z-score (BMIz), WHtR, CRF, MF, SAF, GFS, math, language (Lang), science (Sci), academic achievement average (AAA), and SVI. Finally, a range of 920–951 school children was included based on the mediation analysis.

The total sample size and power calculations were based on the total enrolment of schoolchildren in the Valparaiso region (5th to 8th grades) indicated by the Chilean Ministry of Education in the year 2016 (universe *N* = 951,962). It was considered an alpha error of 5%, a confidence interval of 99%, heterogeneity of 50%, and a 20% dropout. Hence, a total of 797 participants was necessary to reach a representative sample size from the second most populated region in Chile.

### Measurements

School children were evaluated at the school in two four-hour sessions, separated by eight days. Anthropometric measurements (body weight, height, and waist circumference) were evaluated in the first session, and physical fitness was assessed in the second session. Trained instructors from our research team evaluated all measurements. Academic achievement variables were obtained from each student’s school.

### Fatness indicators

The general and unspecific adiposity indicators (i.e., BMIz) were calculated using the World Health Organization’s 2007 growth reference for school-aged children (34). Height was measured using a SECA 213 portable stadiometer (Hamburg, Germany), with the head in the Frankfort plane with a precision of 0.1 cm, and weight was measured with an OMRON (HN-289-LA, Kyoto, Japan) digital scale with a precision of 0.1 kg.

The central adiposity indicator (i.e., WHtR) was obtained by measuring the waist circumference with an inextensible tape (Lufkin, Apex, NC, USA) at the mid-axillary line, at the midpoint between the costal margin and the iliac crest. The result was divided by height to obtain the waist-to-height ratio (waist[cm]/height [cm]).

### Physical fitness assessment

#### Cardiorespiratory fitness

CRF was evaluated through the 20-m shuttle run test (35). Briefly, children ran 20 m from one line to the next for an audio signal, and the intervals between audio signals were reduced each minute. The test ended when children were unable to reach the line twice or felt fatigued. As recommended, the number of completed stages and total time (in seconds) were registered (36). The total time (seconds) was based on age and sex to create a normalized *Z*-score CRF. Appropriate sportswear was suggested to perform this test, and it was held during the morning (between 9:30 and 12:00) in an indoor gym or sports field. The instructors gave verbal instructions about how to perform the test and a brief demonstration of the technique to ensure correct test execution. Adolescents could practice the test and begin when they felt confident.

#### Muscular fitness

MF was obtained after considering upper and lower limb strength. The maximum result of the handgrip strength test was used to determine upper limb strength using a dynamometer (Jamar Plus + Digital Hand Dynamometer, Sammons Preston, USA). The instrument allowed 0 to 90 kg measurements with a 0.1 kg precision and was adjusted to the schoolchildren’s hand size. The procedure to evaluate it was to stand up with the elbow completely extended; the instrument should be pressed firmly with one hand and then with the opposite hand, two attempts per hand, and the maximum score would be registered. The score (kg) was divided by body weight to create a relative measure of this indicator.

A standing long jump test was used to assess lower limb strength. The test consists of standing behind a previously marked line. The instructors give a verbal signal, and the schoolchildren must jump as far as possible and use both feet. This test was performed twice, resting for at least one minute between them. The maximum score was registered in centimeters (cm). The MF score was calculated by adding the standardized *Z*-scores of both tests (adjusted for age and sex).

#### Speed, agility, fitness

Speed, Agility Fitness was evaluated using the 4 × 10 shuttle run test. Movement, agility, and coordination were involved in this test. The application of this test is obtained by demarcating two lines (five meters long) separated by 10 m; additionally, two cones were positioned on each line. Each participant was asked to run as fast as possible from the first line, pick up a piece of cloth located ∼50 cm after the first line, and carry it to the following line. Then they had to leave the cloth, pick up another one, and run to the opposite line. Each schoolchild had two chances to perform the test; the best performance was used, registered in seconds, and multiplied by −1. The SAF *Z*-score was adjusted for age and sex.

#### Global fitness score

The global fitness score (GFS) was obtained by calculating the three main components of the ALPHA fitness test (i.e., CRF, MF, and SAF) (35). Each component was standardized (*Z*-score), and all scores were adjusted for sex and age, with all three added. The procedure for each test is detailed as follows.

### Academic achievement

According to the school records, academic achievement was established through three school subjects (i.e., Math, Lang, and Sci) at the end of the school year. In Chile, the grade scoring range is between 1 and 7 points, and the three subjects are the main subjects included in the Chilean education quality agency evaluation system (SIMCE) and the Programme for International Student Assessment (PISA) by the Organization for Economic Cooperation and Development (OECD). Grades are expressed on a national scale, ranging from 1 to 7. An average of these three subjects was also computed.

### Confounders

In this study, we sought to reduce bias by adjusting the analyses to relevant confounders, such as sex, peak height velocity (PHV), participants’ schools, and SVI. Sex is considered an important moderator in this discipline because visceral fat may affect girls more than boys (37). PHV is a maturity status indicator calculated by subtracting PHV age from chronological age (38). Thus, the maturity offset value is determined by the years of difference between them. Socioeconomic status is also a powerful predictor of various domains, such as academic and neurocognitive performance (39); however, in Latin American countries, a scholar indicator seems to be a better predictor of school achievement than a family SES factor (40). Therefore, we used the SVI, an indicator that measures the socioeconomic vulnerability of students in public/subsidized schools. It ranges from 0 to 100%, with the higher value implying a higher school’s vulnerability. Private schools have a value of 0. SVI is calculated by the Chilean National Board of School Aid and Scholarships (JUNAEB) annually, and it integrates both personal and family indicators (educational level of parents-guardians, SES, students’ health status, physical and emotional well-being, and the school’s geographic location) (41). Finally, the participants’ school was included as a confounder because we assume that each school has certain differences in grading its students.

### Statistical analysis

Table 1 presents the participant characteristics as the mean and standard deviations (SD). The following parametric tests (independent t-student, correlations, and mediations) were used in this study according to the central limit theorem for sample sizes of over 500 participants (42). Simultaneously, a Q-Q plot (quantile-quantile plot) was used to check normality visually. A correlation matrix between fatness, fitness variables, and academic achievements was performed using Pearson’s correlation (Table 2). In addition, multicollinearity was checked before performing mediation analyses (VIF ranged from 1.027 to 1.254), and due to the high rate of participation and representativeness, missing data were not imputed.

**TABLE 1:** Participant characteristics by sex.

		All		Boys		Girls		
	*N*	Mean ± SD	*N*	Mean ± SD	*n*	Mean ± SD	*P*-value	Effect size
Age (years)	1,296	11.89 ± 1.19	648	11.83 ± 1.17	648	11.94 ± 1.21	0.089	0.09
Body Weight (kg)	1,280	50.90 ± 12.04	644	50.03 ± 12.23	636	51.79 ± 11.79	**0.009**	0.15
Height (cm)	1,280	153.04 ± 9.35	644	152.99 ± 10.43	636	153.09 ± 8.13	0.845	0.01
BMI (*Z*-score)	1,280	1.02 ± 1.07	644	1.05 ± 1.11	636	0.99 ± 1.02	0.315	0.06
WHtR	1,250	0.46 ± 0.06	628	0.46 ± 0.05	622	0.45 ± 0.05	< **0.001**	0.23
CRF (*Z*-score)	1,040	0.00 ± 1.00	526	0.00 ± 1.00	514	−0.00 ± 1.00	0.999	0.00
MF (*Z*-score)	1,049	0.03 ± 1.68	521	0.03 ± 1.73	528	0.02 ± 1.62	0.919	0.01
SA-F (*Z*-score)	1,052	0.00 ± 1.00	525	0.00 ± 1.00	527	0.00 ± 1.00	1.000	0.00
GFS	979	0.01 ± 3.10	490	0.01 ± 3.22	489	0.02 ± 2.99	0.951	0.00
Math (grade)	1,275	5.36 ± 0.96	641	5.31 ± 0.95	634	5.41 ± 0.96	0.067	0.10
Language (grade)	1,276	5.40 ± 0.79	641	5.27 ± 0.79	635	5.54 ± 0.77	< **0.001**	0.35
Science (grade)	1,274	5.45 ± 0.84	640	5.32 ± 0.83	634	5.58 ± 0.82	< **0.001**	0.31
AAA	1,276	5.40 ± 0.76	641	5.30 ± 0.75	635	5.51 ± 0.76	< **0.001**	0.28
PHV	1,280	−0.42 ± 1.27	644	−1.17 ± 1.00	636	0.35 ± 1.04	< **0.001**	1.49
SVI	1,296	55.11 ± 35.35	648	57.21 ± 34.17	648	53.02 ± 36.40	**0.033**	0.12

SD: standard deviation; BMI: body mass index; WHtR: waist-to-height ratio; CRF: cardiorespiratory fitness; MF: muscular fitness; SAF: speed-agility fitness; GFS: global fitness score; AAA: academic achievement average; PHV: peak height velocity; SVI: school vulnerability index. Bold values indicate statistical significance. Effect size: Cohen’s d.

**TABLE 2:** Correlation matrix between fatness, fitness, and academic achievement variables.

	BMIz	WHtR	CRF	MF	SAF	GFS	Maths	Lang	Sci	AAA
WHtR	0.830***									
CRF	−0.344***	−0.357***								
MF	−0.458***	−0.517***	0.565***							
SAF	−0.217***	−0.257***	0.497***	0.559***						
GFS	−0.430***	−0.477***	0.790***	0.909***	0.787***					
Maths	−0.130***	−0.120***	0.196***	0.106***	0.107***	0.158***				
Lang	−0.102***	−0.122***	0.146***	0.059	0.022	0.085**	0.659***			
Sci	−0.093***	−0.109***	0.181***	0.105***	0.100**	0.148***	0.688**	0.656***		
AAA	−0.123***	−0.130***	0.198***	0.104***	0.086**	0.148***	0.900***	0.864***	0.883***	
SVI	0.121***	0.181***	−0.178***	−0.231***	−0.129***	−0.225***	−0.116***	−0.098***	−0.126***	−0.129***

BMIz: body mass index (*Z*-score); WHtR: waist-to-height ratio; CRF: cardiorespiratory fitness; MF: muscular fitness; SAF: speed-agility fitness; GFS: global fitness score; Maths; Lang: language; Sci: science; AAA: academic achievement average; SVI: school vulnerability index.

**p < 0.01, ***p < 0.001 indicate statistical significance.

**FIGURE 1:**
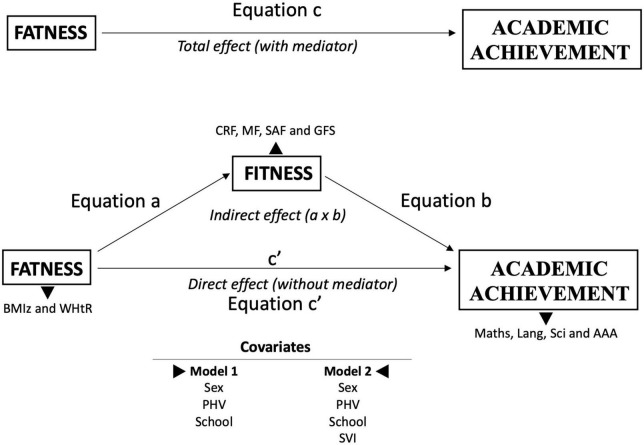
Theoretical approach and general mediation model. BMIz, body mass index (*Z*-score); WHtR, waist-to-height ratio; CRF, cardiorespiratory fitness; MF, muscular fitness; SAF: speed-agility fitness; GFS, global fitness score; sex; PHV, peak height velocity; school; SVI, school vulnerability index.

The mediation effect of fitness indicators on the association between BMIz and WHtR with academic achievements was analyzed using the PROCESS SPSS script (43) through linear regression analysis with bootstrapping (5,000 samples) (44). The theoretical approach and general mediation model are presented in Figure 1. The original four steps proposed originally by Baron and Kenny (45) were considered, step (1) the predictor must significantly predict the outcome variable; step (2) the predictor must significantly predict the mediator; step (3) the mediator must significantly predict the outcome variable, and step (4) the predictor variable must predict the outcome variable less strongly in equation c’ than in equation c. Overall, mediation analysis was performed considering predictors (fatness: BMIz and WHtR), mediators (fitness: CRF, MF, SAF, or GFS), outcome (academic achievements: Math, Lang, Sci, and AAA), and two models (confounding). The general mediation model was structured as follows: equation (a) consisted of the predictor by the mediator; equation (b) was defined as a mediator by the outcome; equation (c) consisted of the predictor by the outcome; and finally, equation (c’) consisted of the predictor and mediator by the outcome. Two models were performed to test our objectives: Model 1: adjusted for sex, PHV, and schools; and Model 2: adjusted for sex, PHV, schools, and SVI. The latter was established to explore the influence of SVI on the central model.

The indirect effects (equation a by equation b) and confidence interval (CI) were established to define a mediation effect (CI not including zero). The percentage of mediation was estimated as 1- (equation c’/equation c). A detailed set of findings (equation a, b, c, c’,% mediation, and category) is presented as Supplementary material (Supplementary Tables 1, 2). The mediation was classified, according to Nitzl et al. (46), as (a) “indirect-only” (full mediation): the indirect effect only exists through the mediator, which means the indirect effect exists but has no direct effect; (b) “complementary” (Partial mediation): a portion of the effect of the predictor on the outcome variable is mediated through the mediator, whereas the predictor still explains a portion of the outcome variable that is independent of a mediator, which means that the indirect and direct effects exist and point in the same direction; (c) “Competitive” (Partial mediation): the same as the complementary classification, both the indirect and direct effects exist but point in different directions; (d) “direct-only” (no mediation): the direct effect exists, but there is no indirect effect, and (e) “no effect” (no mediation): neither the direct nor indirect effect exists (47). For all analyses, the significance level was set at *p* < 0.05.

## Results

The characteristics of participating adolescents and differences by sex are summarized in Table 1. Overall, significant differences between boys and girls were observed in body weight, PHV, WHtR, SVI, Lang, Sci, and AAA. However, no interaction by sex was observed (sex*fatness *p* = 0.603 and sex*fitness *p* = 0.138).

The correlation matrix between all study variables is presented in Table 2. Overall, fatness indicators were negatively correlated with fitness indicators. Academic achievement scores were positively associated with fitness indicators and negatively associated with fatness. Almost all correlations were statistically significant (*p* < 0.05), except for Lang, with MF and SAF (*p* = 0.058 and *p* = 0.472, respectively).

Table 3 shows a summary of all mediation analyses. Overall, it is possible to observe variations in each meditation percentage and some changes in the mediation classification according to models 1 and 2 (without/with SVI as a confounding factor, respectively).

**TABLE 3:** Findings summary concerning the direct and indirect effects according to both models. Percentage of mediation and classification.

		Maths	Language	Science	AAA
**CRF**	**BMIz**	39.1%* → 38.4%* = Δ −**0.7**	37.9%* → 37.2%* = Δ −**0.7**	**51.5% → 52.7%** = Δ + **1.2**	42.4%* → 42.1%* = Δ −**0.3**
		Complementary → Complementary (*n* = 1,022)	Complementary → Complementary (*n* = 1,022)	Direct Only → Direct Only (*n* = 1,021)	Complementary → Complementary (*n* = 1,022)
	**WHtR**	46.7%* → 48.6%* = Δ + **1.8**	30.4%* → 30.2%* = Δ −**0.2**	49.7%* → 53.5%* = Δ + **3.8**	42.7%* → 44.3%* = Δ + **1.6**
		Complementary → Indirect Only (*n* = 1,001)	Complementary → Complementary (*n* = 1,001)	Complementary → Indirect Only (*n* = 1,000)	Complementary → Complementary (*n* = 1,001)
**MF**	**BMIz**	**19.7% → 14.6%** = Δ −**5.2**	**8.8% → 3.6%** = Δ −**5.2**	34.9%* → 29.7 = Δ −**5.2**	**21.8% → 16.4%** = Δ −**5.5**
		Direct Only → Direct Only (*n* = 1,036)	Direct Only → Direct Only (*n* = 1,036)	Indirect Only → No Effect (*n* = 1,034)	No Effect → Direct Only (*n* = 1,036)
	**WHtR**	**20.8% → 16.3%** = Δ −**4.5**	−**0.8% → –6.3%** = Δ + **5.5**	**30.4% → 26.8%** = Δ −**3.6**	**17.8% → 12.8%** = Δ −**5.0**
		Direct Only → Direct Only (*n* = 1,015)	Direct Only → Direct Only (*n* = 1,015)	Direct Only → No Effect (*n* = 1,013)	Direct Only → Direct Only (*n* = 1,015)
**SAF**	**BMIz**	13.7%* → 12.7* = Δ −**1.1**	**1.6% →**−**0.3%** = Δ −**1.9**	18.7%* → 17.9%* = Δ −**0.8**	11.3%* → 10.2% = Δ −**1.2**
		Complementary → Complementary (*n* = 1,032)	Direct Only → Direct Only (*n* = 1,032)	Complementary → Complementary (*n* = 1,030)	Complementary → Direct Only (*n* = 1,032)
	**WHtR**	15.6%* → 15.6%* = Δ **0.0**	**−0.5% → –2.1%** = Δ **–1.6**	18.2%* → 18.7%* = Δ + **0.6**	**10.8% → 10.1%** = Δ −**0.7**
		Complementary → Complementary (*n* = 1,011)	Direct Only → Direct Only (*n* = 1,011)	Complementary → Complementary (*n* = 1,009)	Direct Only → Direct Only (*n* = 1,011)
**GFS**	**BMIz**	38.5%* → 35.4%* = Δ **–3.2**	**23.6% → 19.4%** = Δ **–4.2**	**58.5% → 57.1%** = Δ **–1.5**	40.0%* → 36.7%* = Δ −**3.3**
		Complementary → Complementary (*n* = 967)	Direct Only → Direct Only (*n* = 967)	Direct Only → Direct Only (*n* = 966)	Complementary → Complementary (*n* = 967)
	**WHtR**	45.6%* → 45.4%* = Δ **–0.1**	**13.1% → 9.1%** = Δ **–4.0**	56.5%* → 59.2%* = Δ + **2.7**	38.1%* → 36.9%* = Δ −**1.3**
		Complementary → Indirect Only (*n* = 947)	Direct Only → Direct Only (*n* = 947)	Indirect Only → Indirect Only (*n* = 946)	Complementary → Complementary (*n* = 947)

General scheme: Model 1 → Model 2 = Variation on mediation (%); Model 1: Adjusted to sex, PHV and school; Model 2: Adjusted model 1 + SVI. BMIz: body mass index SD; WHtR: waist-to-height ratio; CRF: cardiorespiratory fitness; MF: muscular fitness; SAF: speed-agility fitness; GFS: global fitness score; Math; Language; Science; AAA: academic achievement average. Mediation and non-mediation type: (a) “Complementary” (mediation): indirect and direct effect exists and points in the same direction; (b) “Competitive” (mediation): indirect and direct effect exists, but in opposite directions; (c) “Indirect-only” (mediation): indirect effect exists, but no direct effect; (e) “Direct-only” (non-mediation): direct effect exists, but no indirect effect; and (f) “No effect” (non-mediation): neither direct nor indirect effect exists.

Overall, a mediation role of CRF, SAF, and GFS is observed, but not of MF. Additionally, the SVI inclusion in the model (model 2) generates significant variations in the mediation percentage and classification.

In particular, the association between BMIz and math, CRF, SAF, and GFS presented “complementary” mediation (models 1 and 2). For Lang, only CRF showed a complementary mediation (models 1 and 2); no mediation was observed for MF, SAF, and GFS. For Sci, only SAF presents a “complementary” mediation (models 1 and 2). Finally, AAA, CRF, and GFS showed “complementary” mediation (models 1 and 2), but SAF lost its mediation role in model 2. In general, no mediation role was observed for MF, and the percentage of mediations ranged from 12.7 to 42.4%, where CRF was the strongest mediator between BMIz and academic achievements.

For the association between WHtR and math, CRF, SAF, and GFS presented “complementary” mediation in model 1; only SAF maintained its mediation after adjusting for SVI (model 2). For Lang, only CRF showed a complementary mediation (models 1 and 2); no mediation was observed for MF, SAF, and GFS (models 1 and 2). With Sci, both CRF and GFS presented an “indirect only” mediation (full mediation, even after adjusting to SVI), and SAF showed a “complementary” mediation role (models 1 and 2). Finally, AAA, CRF, and GFS showed “complementary” mediation (models 1 and 2). In general, no mediation role was observed for MF, and the percentage of mediations ranged from 15.6 to 59.2%, where CRF was the strongest mediator between WHtR and academic achievements. A complete description of all mediation indicators (equations a, b, c, and c’, and others) is available as Supplementary material (Supplementary Tables 1, 2).

## Discussion

This study aimed to determine the mediating role of fitness indicators (CRF, MF, SAF, and GFS) between two fatness indicators related to fat distribution (BMIz and WHtR) and main academic achievements at the national and international levels (math, language, science, and average), as well as to explore the influence of a strong SES factor, such as SVI.

The primary finding confirmed the significant mediating role of fitness in the relationship between fatness and academic achievement. The secondary results showed that (a) CRF, SAF, and GFS had a significant mediation role in the inverse association between fatness and academic achievement, but MF did not; (b) CRF seems to be the most relevant mediator; (c) differences between fatness indicators were observed, in which WHtR obtained the highest percentage of mediation by fitness indicators; and (d) SVI was able to significantly modify both percentages and mediation classifications.

### Differences in fitness component mediations

Presently, the positive association between physical fitness and academic achievement is well established in the literature (21, 48, 49). In this sense, the present findings confirm the evidence indicating that all fitness components (MF, CRF, and SAF) play a certain role in higher academic achievement (21, 48, 50) and that CRF seems to be associated more strongly with educational outcomes than with the other fitness components (49, 50). Indeed, our results align with mediation approaches evidencing the mediation role of CRF in reducing the negative association between fatness and academic achievement (9, 23). However, contrary to some evidence indicating that MF would positively correlate with academic achievement (50, 51), our findings did not display any MF mediation influence on academic achievement according to BMIz and WHtR predictors.

In particular, several studies have shown the short- and long-term effects of aerobic exercise and its physiological marker (CRF) on brain health indicators (i.e., cognitive performance, structural and functional brain activity, the release of brain-derived neurotrophic factor, mood, etc.) (52). However, there are few experimental studies on MF in children’s brains, and the evidence is inconclusive (18). Thus, based on the current evidence, CRF may play a central role in this research area. Supporting this idea and the high degree of interdependence between fitness components, a study in overweight and obese children found that the significant association of MF and SAF with academic achievements weakened after controlling for CRF (50).

Transferring these findings to the educational and public health arenas, we have shown several interventions that have shown the positive impact that physical activity has on the academic achievement of schoolchildren (53, 54) and, likewise, on their cognitive performance (55). Moreover, considering the time children spend in schools, systematic and meta-analytic reviews showed that physical activity at school and physical education classes is positively associated with academic achievement, classroom behavior, and skills related to math and reading, among others (56, 57). Therefore, the recurrent strategy of reducing physical education class time to increase time for math and language subjects could negatively affect adolescent health and educational outcomes (58). Thus, it is recommended to maintain or increase an active lifestyle and, in particular, CRF in schoolchildren to improve factors contributing to academic achievement.

### Differences in fatness indicators related to fat distribution

A key contribution of the present study is to have studied a general adiposity indicator (BMIz) and a central adiposity indicator (WHtR) to establish their differential association with academic achievement when mediated by physical fitness. Our findings showed that WHtR seems more sensitive than BMIz and is affected significantly by physical fitness indicators, mainly CRF. Thus, our results support the importance of studying fatness indicators related to its distribution because the mediation role of fitness would depend on them (14).

In this sense, our findings displayed a differential association between the two fat indicators analyzed in this study. This could be because BMIz is considered a surrogate and unspecific obesity marker (it does not differentiate between peripheral and central obesity), while WHtR seems more specific and more substantially related to inflammation and cognitive functioning (14, 16). The main rationale behind the above is that excess adiposity in childhood has been linked to higher low-level inflammation, which has been associated with the development of neurodegeneration in adulthood (16, 59). Thus, children and adolescents living with obesity have shown reduced gray matter in the prefrontal cortex, lower cognitive performance, and reduced academic achievement (2). Therefore, preventing fat storage in more specific zones related to higher inflammation markers throughout the body is crucial to promoting healthy development in childhood.

To achieve this goal, physical activity and improved physical fitness are fundamental. For instance, in children and adolescents with obesity, exercise is more effective than diet alone or in combination with diet and exercise to reduce visceral fat (60, 61). In turn, a cross-sectional and longitudinal analysis showed that children with or improving their CRF showed lower inflammation levels (high-sensitivity C-reactive protein), regardless of their body composition (62). In addition, a network meta-analysis showed that high-intensity interval training and aerobic exercise were the most effective strategies to reduce visceral fat compared to strength exercise (63). In this way, the mediation role of CRF, SAF, and GFS in this study could be related mainly to the influence or shared participation of CRF on these fitness components and the global indicator. The main reason for speculating this is that CRF has been deemed the primary predictor of maximal fat oxidation (64).

### Exploring the influence of an school vulnerability index

A novel approach in this study was to explore a complex SES indicator related to adolescents’ vulnerability in school and establish its implications in the relationship between fatness, fitness, and academic achievement. It is pertinent to carry out this type of methodology because most of the academic differences among schoolchildren can be explained by their social background (27, 65). Thus, including this SES variable in a second statistical model gives us an overview of a) the influence of SES on this set of mediation analyses and b) the relevance of fitness as a mediator if it keeps its statistical significance, even after controlling for SVI.

Overall, we found that SVI showed a differential influence according to the model analyzed, generating greater variation (percentages and classification mediations) in WHtR than BMIz. This scenario supports our study aim due to this statistical approach’s higher specificity of WHtR than BMIz. In addition, it reinforces that both CRF and GFS play a relevant role regardless of adolescents’ school vulnerability influence.

These findings become significant if we consider two main points. First is the high dependence of fatness, fitness, and academic achievement on SES (65–67). Second, the worldwide prevalence of fatness; the secular trend indicating a reduction in physical fitness in children and adolescents; the global concern for educational achievement in low- and middle-income countries; and the health, economic, and educational effects of the COVID-19 pandemic (68–70). In this sense, and addressing a close area linked to academic achievement, a recent study has shown that adolescents from schools with a high vulnerability index and a high fitness level present better cognitive performance than their unfit peers (41). In addition, this fit group had no statistical differences compared to their unfit peers from schools with a lower vulnerability index (41). Therefore, the present study supports the current literature in this field (14, 41), suggesting that having better physical fitness could act as a social protective factor related to bridging the gap at the academic level derived from school vulnerability. Experimental and longitudinal studies are needed to corroborate this assumption.

### Limitations and strengths

The main limitation of the present study is its cross-sectional design, which reduces the possibility of determining causality among variables. In addition, fitness and fatness indicators were evaluated by field-based and indirect tests, respectively, which could increase methodological biases; nonetheless, it is feasible to implement these measurements in school settings. Finally, the academic evaluation depends on the school’s increasing biases.

The main strengths are the large sample size of adolescents from a Latin American country and the inclusion of several fatness and fitness indicators, improving understanding in this research area. Moreover, we analyzed the three main subjects evaluated internationally (PISA). Finally, we explored the influence of a powerful SES indicator, such as the SVI, which allowed us to determine a novel finding concerning the mediator role of fitness, regardless of the adolescents’ social background profile.

## Conclusion

In conclusion, a higher level of global physical fitness, but mainly CRF, mediates the detrimental influence of fatness on adolescent academic achievement. This favorable influence was shown to be constant, even for a central fatness indicator such as WHtR and a critical socioeconomic factor. Indeed, our findings reinforce the relevant role of CRF and GFS, regardless of adolescents’ school vulnerability influence. This is crucial at a public health level, considering the strong relationship between obesity and adolescent socioeconomic background. Thus, these findings become essential if we consider the current high rate of obesity, low educational and fitness performance in childhood, and the increasing poverty rate globally in low- to middle-income countries. Therefore, governments should create public policies that encourage physical activity, focusing on enhancing adolescents’ physical fitness. Experimental and longitudinal studies in this research area are warranted.

## Data availability statement

The datasets presented in this article are not readily available because of ethical restrictions. Requests to access the datasets should be directed to CC-M, carlos.cristi.montero@gmail.com.

## Ethics statement

The studies involving human participants were reviewed and approved by Ethics Committee of the Pontificia Universidad Católica de Valparaíso (BIOEPUCV-H103–2016). Written informed consent to participate in this study was provided by the participants’ legal guardian/next of kin.

## Author contributions

CC-M contributed to the design of the project. CC-M and GG-A conceptualized the design of the study, analyzed the data, and wrote the concept version of the manuscript. SH-J, JO-A, GF, and PD-F critically reviewed the manuscript and edited the article. All authors have given final approval of the manuscript and agreed to be accountable for the accuracy and integrity of any part of the work.
