# Supplementary material for: Physical fitness mediates the inverse association between fatness indicators and academic achievement, despite the school vulnerability of adolescents—The Cogni-Action Project
Source: Front Nutr. 2022 Oct 26;9:904831. doi: 10.3389/fnut.2022.904831 (PMC9643798; doi:10.3389/fnut.2022.904831)
Supplement: Supplementary file 1 [file Data_Sheet_1.pdf]

Supplementary material

Table S1, Mediation analysis between BMIz and academic achievements mediated by fitness.

		n	Predictor - Mediator a	Mediator – Outcome b	Total effect c	Direct effect c'	Indirect effect a x b	Mediation %	Mediation Partial/Full
BMIz-CRF-Math	Model 1	1022	0.2 (-0.37, -0.27)	0.16 (0.1, 0.22)	-0.13 (-0.19, -0.08)	-0.08* (-0.14, -0.02)	-0.05* (-0.08, -0.03)	39,10%	Complementary
	Model 2	1022	-0.31 (-0.36, -0.25)	0.154 (0.092, 0.215)	-0.12 (-0.18, -0.07)	-0.075* (-0.132, -0.0186)	-0.047* (-0.069, -0.027)	38,40%	Complementary
BMIz-CRF-Lang	Model 1	1022	-0.32 (-0.37, -0.27)	0.096 (0.046, 0.147)	-0.08 (-0.13, -0.04)	-0.05* (-0.097, -0.004)	-0.03* (-0.05, -0.014)	37,90%	Complementary
	Model 2	1022	-0.31 (-0.36, -0.25)	0.091 (0.04, 0.141)	-0.07 (-0.12, -0.03)	-0.047* (-0.094, -0.0003)	-0.0278* (-0.044, -0.012)	37.2%	Complementary
BMIz-CRF-Sci	Model 1	1021	-0.32 (-0.37, -0.27)	0.14 (0.08, 0.19)	-0.08 (-0.13, -0.04)	-0.04 (-0.09, 0.01)	-0.04* (-0.06, -0.03)	51.5%	Indirect Only
	Model 2	1021	-0.31 (-0.36, -0.25)	0.127 (0.073, 0.18)	-0.07 (-0.12, -0.03)	-0.035 (-0.084, 0.0148)	-0.0387* (-0.058, -0.021)	52.7%	Indirect Only
BMIz-CRF-AAA	Model 1	1022	-0.32 (-0.37, -0.27)	0.13 (0.08, 0.18)	-0.1 (-0.14, -0.06)	-0.06* (-0.1, -0.01)	-0.04* (-0.06, -0.03)	42.4%	Complementary
	Model 2	1022	-0.31 (-0.36, -0.25)	0.124 (0.075, 0.172)	-0.09 (-0.13, -0.05)	-0.052* (-0.097, -0.0071)	-0.0378* (-0.055, -0.022)	42.1%	Complementary
BMIz-MF-Math	Model 1	1036	-0.71 (-0.8, -0.63)	0.03 (0. 0.07)	-0.13 (-0.18, -0.07)	-0.1* (-0.16, -0.04)	-0.02 (-0.05, 0.003)	19.7%	Direct Only
	Model 2	1036	-0.68 (-0.76, -0.59)	0.024 (-0.015, 0.063)	-0.11 (-0.17, -0.06)	-0.096* (-0.157, -0.0361)	-0.0163 (-0.045, 0.011)	14.6%	Direct Only
BMIz-MF-Lang	Model 1	1036	-0.71 (-0.8, -0.63)	0.01 (-0.02, 0.04)	-0.08 (-0.12, -0.04)	-0.07* (-0.12, -0.02)	-0.01 (-0.03, 0.02)	8.8%	Direct Only
	Model 2	1036	-0.68 (-0.76, -0.59)	0.004 (-0.028, 0.036)	-0.07 (-0.12, -0.03)	-0.07* (-0.12, -0.0207)	-0.0027 (-0.026, 0.02)	3.6%	Direct Only
BMIz-MF-Sci	Model 1	1034	-0.71 (-0.8, -0.63)	0.039 (0.005, 0.072)	-0.08 (-0.13, -0.03)	-0.05 (-0.104, 0.001)	-0.028* (-0.05, -0.003)	34.9%	Indirect Only
	Model 2	1034	-0.68 (-0.76, -0.59)	0.03 (-0.004, 0.064)	-0.07 (-0.11, -0.02)	-0.047 (-0.1, 0.005)	-0.02 (-0.044, 0.003)	29.7%	No Effect
BMIz-MF-AAA	Model 1	1036	-0.71 (-0.8, -0.63)	0.029 (-0.002, 0.06)	-0.09 (-0.14, -0.05)	-0.07* (-0.12, -0.03)	-0.02 (-0.04, 0.002)	21.8%	No Effect
	Model 2	1036	-0.68 (-0.76, -0.59)	0.02 (-0.011, 0.051)	-0.08 (-0.13, -0.04)	-0.07* (-0.118, -0.0222)	-0.0137 (-0.035, 0.009)	16.4%	Direct Only
BMIz-SAF-Math	Model 1	1032	-0.2 (-0.26, -0.15)	0.08 (0.03, 0.14)	-0.12 (-0.18, -0.07)	-0.11* (-0.16, -0.05)	-0.02* (-0.03, -0.01)	13.7%	Complementary
	Model 2	1032	-0.19 (-0.24, -0.13)	0.074 (0.015, 0.133)	-0.11 (-0.16, -0.06)	-0.096* (-0.152, -0.0413)	-0.014* (-0.027, -0.003)	12.7%	Complementary
BMIz-SAF-Lang	Model 1	1032	-0.2 (-0.26, -0.15)	0.006 (-0.043, 0.054)	-0.08 (-0.12, -0.03)	-0.07* (-0.119, -0.029)	-0.001 (-0.01, 0.009)	1.6%	Direct Only
	Model 2	1032	-0.19 (-0.24, -0.13)	-0.001 (-0.049, 0.048)	-0.07 (-0.11, -0.02)	-0.068* (-0.113, -0.0223)	0.0001 (-0.009, 0.01)	-0.3%	Direct Only
BMIz-SAF-Sci	Model 1	1030	-0.2 (-0.26, -0.14)	0.07 (0.02, 0.12)	-0.08 (-0.12, -0.03)	-0.06* (-0.11, -0.02)	-0.01* (-0.03, -0.004)	18.7%	Complementary
	Model 2	1030	-0.19 (-0.24, -0.13)	0.063 (0.011, 0.114)	-0.07 (-0.11, -0.02)	-0.054* (-0.101, -0.0057)	-0.0118* (-0.023, -0.002)	17.9%	Complementary
BMIz-SAF-AAA	Model 1	1032	-0.2 (-0.26, -0.15)	0.051 (0.005, 0.098)	-0.09 (-0.13, -0.05)	-0.08* (-0.12, -0.04)	-0.01* (-0.02, -0.001)	11.3%	Complementary
	Model 2	1032	-0.19 (-0.24, -0.13)	0.043 (-0.004, 0.09)	-0.08 (-0.12, -0.04)	-0.072* (-0.116, -0.0287)	-0.0081 (-0.018, 0.001)	10.2%	Direct Only
BMIz-GFS-Math	Model 1	967	-1.25 (-1.42, -1.09)	0.04 (0.02, 0.06)	-0.13 (-0.18, -0.07)	-0.08* (-0.14, -0.02)	-0.05* (-0.08, -0.02)	38.5%	Complementary
	Model 2	967	-1.19 (-1.35, -1.02)	0.034 (0.013, 0.055)	-0.11 (-0.17, -0.06)	-0.074* (-0.135, -0.0124)	-0.0403* (-0.067, -0.015)	35.4%	Complementary
BMIz-GFS-Lang	Model 1	967	-1.25 (-1.42, -1.09)	0.015 (-0.003, 0.032)	-0.08 (-0.12, -0.03)	-0.06* (-0.11, -0.01)	-0.02 (-0.04, 0.004)	23.6%	Direct Only
	Model 2	967	-1.19 (-1.35, -1.02)	0.011 (-0.006, 0.029)	-0.07 (-0.11, -0.02)	-0.056* (-0.106, -0.0056)	-0.0134 (-0.036, 0.009)	19.4%	Direct Only
BMIz-GFS-Sci	Model 1	966	-1.25 (-1.42, -1.08)	0.04 (0.02, 0.05)	-0.08 (-0.13, -0.03)	-0.03 (-0.09, 0.02)	-0.04* (-0.07, -0.02)	58.5%	Indirect Only
	Model 2	966	-1.19 (-1.35, -1.02)	0.031 (0.013, 0.05)	-0.07 (-0.11, -0.02)	-0.028 (-0.081, 0.0257)	-0.0371* (-0.06, -0.015)	57.1%	Indirect Only
BMIz-GFS-AAA	Model 1	967	-1.25 (-1.42, -1.09)	0.03 (0.01, 0.05)	-0.09 (-0.14, -0.05)	-0.06* (-0.1, -0.01)	-0.04* (-0.06, -0.02)	40.0%	Complementary
	Model 2	967	-1.19 (-1.35, -1.02)	0.025 (0.009, 0.042)	-0.08 (-0.13, -0.04)	-0.052* (-0.101, -0.0035)	-0.0302* (-0.051, -0.01)	36.7%	Complementary

*Model 1: Adjusted to sex, PHV and school; Model 2: Adjusted model 1 + SVI. BMIz: Body Mass Index BMIz: body (Z-score); CRF: cardiorespiratory fitness; MF: muscular fitness; SAF: speed-agility fitness; GFS: global fitness score; Math; Lang: Language; Sci: Science; AAA: Academic Achievement Average. Mediation and non-mediation type: a) "Complementary" (mediation): indirect and direct effect exist and point in the same direction, b) "Competitive" (mediation): indirect and direct effect exist, but in opposite directions, c) "Indirect-only" (mediation): indirect effect exist, but no direct effect, e) "Direct-only" (non-mediation): direct effect exist, but no indirect effect, and f) "No effect" (non-mediation): neither direct nor indirect effect exist. * indicate statistical significance.*

Table S2. Mediation analysis between WHtR and academic achievements mediated by fitness.

		n	Predictor - Mediator<br>a	Mediator – Outcome<br>b	Total effect<br>c	Direct effect<br>c'	Indirect effect<br>a x b	Mediation<br>%	Mediation<br>Partial/Full
WHtR-CRF-Math	Model 1	1001	-6.58 (-7.65, -5.51)	0.17 (0.11, 0.23)	-2.39 (-3.46, -1.31)	-1.27* (-0.14, -1.33)	-1.12* (-1.59, -0.7)	46.7%	Complementary
	Model 2	1001	-6.22 (-7.3, -5.13)	0.161 (0.099, 0.22)	-2.06 (-3.15, -0.96)	-1.06 (-2.2. 0.09)	-0.999* (-1.44, -0.6)	48.6%	Indirect Only
WHtR-CRF-Lang	Model 1	1001	-6.58 (-7.65, -5.51)	0.09 (0.04, 0.14)	-1.88 (-2.75, -1.01)	-1.31* (-2.23, -0.38)	-0.57* (-0.94, -0.23)	30.4%	Complementary
	Model 2	1001	-6.22 (-7.3, -5.13)	0.081 (0.03, 0.13)	-1.66 (-2.54, -0.78)	-1.16* (-2.09, -0.23)	-0.501* (-0.85, -0.18)	30.2%	Complementary
WHtR-CRF-Sci	Model 1	1000	-6.58 (-7.65, -5.51)	0.13 (0.08, 0.19)	-1.74 (-2.68, -0.8)	-0.88 (-1.87. 0.12)	-0.87* (-1.25, -0.49)	49.7%	Complementary
	Model 2	1000	-6.21 (-7.3, -5.13)	0.12 (0.069, 0.18)	-1.43 (-2.38, -0.47)	-0.66 (-1.67. 0.34)	-0.763* (-1.13, -0.4)	53.5%	Indirect Only
WHtR-CRF-AAA	Model 1	1001	-6.58 (-7.65, -5.51)	0.13 (0.08, 0.18)	-1.99 (-2.84, -1.15)	-1.14* (-2.04, -0.25)	-0.85* (-1.22, -0.5)	42.7%	Complementary
	Model 2	1001	-6.22 (-7.3, -5.13)	0.12 (0.073, 0.17)	-1.7 (-2.56, -0.84)	-0.95* (-1.85, -0.05)	-0.754* (-1.11, -0.43)	44.3%	Complementary
WHtR-MF-Math	Model 1	1015	-16.14 (-17.78, -14.5)	0.03 (-0.01, 0.07)	-2.37 (-3.44, -1.29)	-1.88* (-3.13, -0.62)	-0.49 (-1.21. 0.14)	20.8%	Direct Only
	Model 2	1015	-15.39 (-17.04, -13.73)	0.02 (-0.02, 0.06)	-2 (-3.09, -0.91)	-1.68* (-2.93, -0.42)	-0.326 (-0.98. 0.32)	16.3%	Direct Only
WHtR-MF-Lang	Model 1	1015	-16.14 (-17.78, -14.5)	0 (-0.03, 0.03)	-1.87 (-2.75, -1)	-1.89* (-2.91, -0.87)	0.01 (-0.54. 0.55)	-0.8%	Direct Only
	Model 2	1015	-15.39 (-17.04, -13.73)	-0.01 (-0.04, 0.03)	-1.66 (-2.55, -0.77)	-1.76* (-2.79, -0.74)	0.105 (-0.43. 0.63)	-6.3%	Direct Only
WHtR-MF-Sci	Model 1	1013	-16.14 (-17.77, -14.5)	0.03 (0, 0.07)	-1.67 (-2.61, -0.74)	-1.16* (-2.26, -0.07)	-0.51 (-1.07. 0.07)	30.4%	Direct Only
	Model 2	1013	-15.38 (-17.04, -13.73)	0.02 (-0.012, 0.06)	-1.35 (-2.3, -0.4)	-0.99 (-2.09. 0.11)	-0.363 (-0.93. 0.17)	26.8%	No effect
WHtR-MF-AAA	Model 1	1015	-16.14 (-17.78, -14.5)	0.02 (-0.01, 0.05)	-1.97 (-2.82, -1.11)	-1.62* (-2.61, -0.62)	-0.35 (-0.88. 0.19)	17.8%	Direct Only
	Model 2	1015	-15.39 (-17.04, -13.73)	0.01 (-0.019, 0.05)	-1.66 (-2.53, -0.79)	-1.45* (-2.45, -0.45)	-0.212 (-0.72. 0.3)	12.8%	Direct Only
WHtR-SAF-Math	Model 1	1011	-4.68 (-5.79, -3.58)	0.08 (0.02, 0.14)	-2.34 (-3.41, -1.27)	-1.97* (-3.08, -0.87)	-0.37* (-0.66, -0.08)	15.6%	Complementary
	Model 2	1011	-4.39 (-5.52, -3.27)	0.07 (0.01, 0.13)	-1.96 (-3.04, -0.87)	-1.65* (-2.76, -0.53)	-0.306* (-0.59, -0.04)	15.6%	Complementary
WHtR-SAF -Lang	Model 1	1011	-4.68 (-5.79, -3.58)	0 (-0.05, 0.05)	-1.86 (-2.72, -0.99)	-1.86* (-2.76, -0.97)	0.01 (-0.23. 0.26)	-0.5%	Direct Only
	Model 2	1011	-4.39 (-5.52, -3.27)	-0.01 (-0.056, 0.04)	-1.61 (-2.49, -0.73)	-1.64* (-2.55, -0.74)	0.034 (-0.18. 0.28)	-2.1%	Direct Only
WHtR-SAF-Sci	Model 1	1009	-4.67 (-5.77, -3.57)	0.07 (0.01, 0.12)	-1.7 (-2.64, -0.77)	-1.39* (-2.36, -0.43)	-0.31* (-0.58, -0.07)	18.2%	Complementary
	Model 2	1009	-4.37 (-5.49, -3.25)	0.06 (0.006, 0.11)	-1.36 (-2.31, -0.42)	-1.11* (-2.08, -0.14)	-0.255* (-0.51, -0.03)	18.7%	Complementary
WHtR-SAF-AAA	Model 1	1011	-4.68 (-5.79, -3.58)	0.05 (0, 0.09)	-1.96 (-2.81, -1.11)	-1.75* (-2.62, -0.87)	-0.21 (-0.45. 0.02)	10.8%	Direct Only
	Model 2	1011	-4.39 (-5.52, -3.27)	0.04 (-0.01, 0.08)	-1.63 (-2.49, -0.77)	-1.47* (-2.35, -0.58)	-0.165 (-0.38. 0.04)	10.1%	Direct Only
WHtR-GFS-Math	Model 1	947	-27.39 (-30.61, -24.17)	0.04 (0.02, 0.06)	-2.35 (-3.45, -1.25)	-1.28* (-2.53, -0.03)	-1.07* (-1.72, -0.5)	45.6%	Complementary
	Model 2	947	-25.94 (-29.19, -22.69)	0.03 (0.013, 0.06)	-1.97 (-3.09, -0.85)	-1.07 (-2.32. 0.18)	-0.894* (-1.52, -0.34)	45.4%	Indirect Only
WHtR-GFS-Lang	Model 1	947	-27.39 (-30.61, -24.17)	0.01 (-0.01, 0.03)	-1.92 (-2.81, -1.03)	-1.67* (-2.68, -0.66)	-0.25 (-0.75. 0.23)	13.1%	Direct Only
	Model 2	947	-25.94 (-29.19, -22.69)	0.01 (-0.012, 0.02)	-1.68 (-2.58, -0.77)	-1.52* (-2.54, -0.51)	-0.153 (-0.63. 0.32)	9.1%	Direct Only
WHtR-GFS-Sci	Model 1	946	-27.37 (-30.59, -24.15)	0.03 (0.01, 0.05)	-1.63 (-2.6, -0.67)	-0.71 (-1.8. 0.38)	-0.92* (-1.47, -0.41)	56.5%	Indirect Only
	Model 2	946	-25.91 (-29.16, -22.65)	0.03 (0.01, 0.05)	-1.29 (-2.28, -0.31)	-0.53 (-1.62. 0.57)	-0.766* (-1.27, -0.28)	59.2%	Indirect Only
WHtR-GFS-AAA	Model 1	947	-27.39 (-30.61, -24.17)	0.03 (0.01, 0.04)	-1.96 (-2.83, -1.09)	-1.21* (-2.2, -0.23)	-0.75* (-1.23, -0.26)	38.1%	Complementary
	Model 2	947	-25.94 (-29.19, -22.69)	0.02 (0.006, 0.04)	-1.63 (-2.52, -0.75)	-1.03* (-2.02, -0.05)	-0.602* (-1.05, -0.15)	36.9%	Complementary

*Model 1: Adjusted to sex, PHV and school; Model 2: Adjusted model 1 + SVI; WHtR: Waist-to-height ratio; CRF: cardiorespiratory fitness; MF: muscular fitness; SAF: speed-agility fitness; GFS: global fitness score; Math; Lang: Language; Sci: Science; AAA: Academic Achievement Average. Mediation and non-mediation type: a) "Complementary" (mediation): indirect and direct effect exist and point in the same direction, b) "Competitive" (mediation): indirect and direct effect exist, but in opposite directions, c) "Indirect-only" (mediation): indirect effect exist, but no direct effect, e) "Direct-only" (non-mediation): direct effect exist, but no indirect effect, and f) "No effect" (non-mediation): neither direct and indirect effect exist. * indicate statistical significance.*
